# Supplementary material for: Breastfeeding initiation and duration through the COVID-19 pandemic, a linked population-level routine data study: the Born in Wales Cohort 2018–2021
Source: BMJ Paediatr Open. 2023 Jul 11;7(1):e001907. doi: 10.1136/bmjpo-2023-001907 (PMC10347487; doi:10.1136/bmjpo-2023-001907)
Supplement: Author's manuscript [file bmjpo-2023-001907.draft_revisions.pdf]

BMJ Paediatrics Open

BMJ Paediatrics Open is committed to open peer review. As part of this commitment we make the peer review history of every article we publish publicly available.

When an article is published we post the peer reviewers' comments and the authors' responses online. We also post the versions of the paper that were used during peer review. These are the versions that the peer review comments apply to.

The versions of the paper that follow are the versions that were submitted during the peer review process. They are not the versions of record or the final published versions. They should not be cited or distributed as the published version of this manuscript.

BMJ Paediatrics Open is an open access journal and the full, final, typeset and author-corrected version of record of the manuscript is available on our site with no access controls, subscription charges or pay-per-view fees (http://bmjpaedsopen.bmj.com).

If you have any questions on BMJ Paediatrics Open's open peer review process please email info.bmjpo@bmj.com

BMJ Paediatrics Open

Intention to breastfeed and subsequent breastfeeding duration, a linked population-level routine data study - The Born in Wales cohort 2018-2021

Journal:	*BMJ Paediatrics Open*
Manuscript ID	bmjpo-2023-001907
Article Type:	Original research
Date Submitted by the Author:	10-Feb-2023
Complete List of Authors:	Jones, Hope; National Centre for Population Health and Wellbeing Research, Swansea University Medical School, Faculty of Medicine, Health & Life Science<br>Seaborne, Mike; National Centre for Population Health and Wellbeing Research, Swansea University Medical School, Faculty of Medicine, Health & Life Science<br>Mhereeg, Mohamed; National Centre for Population Health and Wellbeing Research, Swansea University Medical School, Faculty of Medicine, Health & Life Science<br>James, Michaela; National Centre for Population Health and Wellbeing Research, Swansea University Medical School, Faculty of Medicine, Health & Life Science<br>Kennedy, Natasha; National Centre for Population Health and Wellbeing Research, Swansea University Medical School, Faculty of Medicine, Health & Life Science<br>Bandyopadhyay, Amrita; National Centre for Population Health and Wellbeing Research, Swansea University Medical School, Faculty of Medicine, Health & Life Science<br>Brophy, Sinead; National Centre for Population Health and Wellbeing Research, Swansea University Medical School, Faculty of Medicine, Health & Life Science
Keywords:	COVID-19, Data Collection

SCHOLARONE™
Manuscripts

I, the Submitting Author has the right to grant and does grant on behalf of all authors of the Work (as defined in the below author licence), an exclusive licence and/or a non-exclusive licence for contributions from authors who are: i) UK Crown employees; ii) where BMJ has agreed a CC-BY licence shall apply, and/or iii) in accordance with the terms applicable for US Federal Government officers or employees acting as part of their official duties; on a worldwide, perpetual, irrevocable, royalty-free basis to BMJ Publishing Group Ltd ("BMJ") its licensees and where the relevant Journal is co-owned by BMJ to the co-owners of the Journal, to publish the Work in this journal and any other BMJ products and to exploit all rights, as set out in our licence.

The Submitting Author accepts and understands that any supply made under these terms is made by BMJ to the Submitting Author unless you are acting as an employee on behalf of your employer or a postgraduate student of an affiliated institution which is paying any applicable article publishing charge ("APC") for Open Access articles. Where the Submitting Author wishes to make the Work available on an Open Access basis (and intends to pay the relevant APC), the terms of reuse of such Open Access shall be governed by a Creative Commons licence – details of these licences and which Creative Commons licence will apply to this Work are set out in our licence referred to above.

Other than as permitted in any relevant BMJ Author's Self Archiving Policies, I confirm this Work has not been accepted for publication elsewhere, is not being considered for publication elsewhere and does not duplicate material already published. I confirm all authors consent to publication of this Work and authorise the granting of this licence.

Confidential: For Review Only

Intention to breastfeed and subsequent breastfeeding duration, a linked population-level routine data study - The Born in Wales cohort 2018-2021

Jones H[1], Seaborne M[1], Mhereeg M[1], James M[1], Kennedy N[1], Bandyopadhyay A[1], Brophy S[1]

Affiliations:

[1]National Centre for Population Health and Wellbeing Research, Swansea University Medical School, Faculty of Medicine, Health & Life Science, Swansea, Wales, UK.

Corresponding Author: h.e.jones@swansea.ac.uk, Hope Eleri Jones, Data Science Building, Swansea University, Swansea, Wales, SA2 8PP

Keywords: Breastfeeding, Intention, Exclusive Breastfeeding, Linked Data

Word count: 2499

Confidential: For Review Only

Abstract

Objective: The World Health Organisation recommends exclusive breastfeeding for the first six months of life. This study aimed to examine 1) If intention to breastfeed is associated with higher levels of breastfeeding, and 2) If the pandemic impacted the intention to breastfeed and the breastfeeding rates in Wales.

Methods: A cohort study utilising routinely collected, linked healthcare data from the Secure Anonymised Information Linkage databank (SAIL). All women that gave birth between 2018 and 2021 recorded in the Maternal Indicators (MIDS) and National Community Child Health (NCCH) Births and Breastfeeding datasets were included.

Results: Intention to breastfeed was associated with being 27.5 times more likely to continue to breastfeed for six months compared to those who did not intend to breastfeed (OR 27.5, 95% CI: 24.8-30.5). 57.8% of expectant mothers intended to breastfeed pre-pandemic and 58.7% intended to breastfeed in 2020. Breastfeeding rates to six months were 16.6% pre-pandemic and 20.5% in 2020. When compared to a survey population, the initial intention to breastfeed/not breastfeed only changes for about 10% of women.

Conclusion: The biggest predictor of breastfeeding at six months was intention to breastfeed. During the pandemic those who intended to breastfeed were more likely to breastfeed for six months compared to before and after the pandemic. Therefore, targeted interventions to encourage intention to breastfeed could improve breastfeeding rates and duration.

Confidential: For Review Only

Introduction

The considerable health and social benefits of breastfeeding are already well-established [1]. Breastfeeding provides both short-term and long-term physical, economic and environmental improvements for young children, women, and society. Benefits to breastfed babies include fewer infections, increased intelligence, and protection against obesity and diabetes. Exclusively breastfed babies have been demonstrated to have a higher intelligence quotient (IQ) and may have higher mathematical abilities than artificially fed babies (those fed with bottle milk, formula milk or alternative methods) [2]. They are also less prone to infections, asthma, other atopy, and allergic diseases. Moreover, breastfeeding is beneficial for mothers as research indicates that it reduces the risk of breast, uterine and ovarian cancer, reduces postpartum bleeding, and is beneficial in promoting postpartum weight loss [2]. Breastfeeding supports both short-term and long-lasting health environmental benefits for infants, mothers, and the wider population (Rollins et al, 2016). To recognise the advantages of breastfeeding, political support and financial investment are essential to protect, promote, and support breastfeeding [3].

The current World Health Organisation (WHO) recommendations indicate that mothers should provide exclusive breastfeeding to their babies during the first six months of life [4]. However, the United Kingdom (UK) has one of the lowest breastfeeding rates in the world. In fact, 80% of babies are breastfed at birth but only 1% are exclusively breastfed for six months in the UK [5]. These breastfeeding rates are lower among women in areas of higher deprivation and health inequalities [6]. Breastfeeding rates in Wales have remained consistent despite investment in services

Confidential: For Review Only

and UNICEF baby friendly initiative accreditation (Welsh Government, 2019). It is acknowledged that this is a multi-faceted issue relating to population health, initiation, and maintenance of breastfeeding. It is important to note that breastfeeding rates are influenced by various socio-economic factors, religion, education, and support services available [6].

Factors that influence women's breastfeeding decisions can include breastfeeding intention, breastfeeding self-efficacy and social support [7]. Exclusive breastfeeding intention describes a mother's intention to provide only breast milk for their baby from birth until at the age of six months. Self-efficacy refers to an individual's belief in their ability to complete the necessary behaviours to achieve specific outcomes. Breastfeeding promotion strategies conducted by midwives often include social support, however, there is minimal research surrounding intention to breastfeed and self-efficacy and whether this impacts future breastfeeding behaviour.

In 2020, the COVID-19 pandemic and concerns about transmission of the disease contributed to higher rates of breastfeeding interruption [8]. Research has found that the risk of transmission from infected mothers to their offspring is extremely low [8]. Furthermore, available data showed that the lack of support for lactating mothers during the pandemic contributed to breastfeeding cessation worldwide [8]. Few strategies were proposed to overcome this issue.

In the year 2020, research found that a third of women who planned to breastfeed reported that they did not receive help with positioning the infant [9]. Additionally, one in four women perceived that they did not get enough support with

Confidential: For Review Only

feeding in the hospital [9]. This could have been due to the increased burden on healthcare systems and pressure placed on healthcare professionals to discharge mothers sooner to minimise infection risks, resulting in fewer opportunities to support mothers with infant feeding. It is unclear whether rates of breastfeeding changed again after the periods of lockdowns and restrictions in Wales and upon returning to normal day-to-day activity.

The protection, promotion, and support of breastfeeding is a priority for public health. Therefore, encouraging optimal breastfeeding practices including breastfeeding initiation within one hour after birth, exclusive breastfeeding for six months, and continuation of breastfeeding for at least two years once nutritious and safe complementary foods are introduced at six months is crucial for meeting the Sustainable Development Goals by 2030 [10]. For example, goals 2 and 3 are concerned with hunger, health, and well-being [10]. Breastfeeding is a vital source of nutrition that can save children’s lives and contribute to improved health outcomes for children and mothers.

This study aimed to examine 1) If intention to breastfeed is associated with higher levels of breastfeeding, and 2) If the pandemic impacted the intention to breastfeed and the breastfeeding rates in Wales. Understanding what factors impact on breastfeeding to the recommended six months will help develop more targeted interventions to promote and support breastfeeding.

Confidential: For Review Only

Methods

Study Design and Setting

This was a cohort study utilising routinely collected anonymised population-level linked data from the Secure Anonymised Information Linkage (SAIL) databank [11,12]. Data sources include the Maternal Indicators (MIDS) and the National Community Child Health (NCCH) Births and Breastfeeding datasets. All women in Wales included in the NCCH Breastfeeding and MIDS data were deemed eligible for analysis; any stillbirths were excluded. In cases where women had multiple reported births, all births were selected as the mother’s outcome of breastfeeding and intention to feed at each birth is independent of the decisions at another birth. Breastfeeding rates were compared from 2018 to 2021.

Data Sources and Linkage

Analysis was undertaken using anonymised population-scale, individual level linked routinely collected national-scale data available in the SAIL Databank [11,12]. This anonymously links a wide range of person-based data using a unique, encrypted personal identifier. The linkage in this study includes the MIDS data, NCCH Births and Breastfeeding data. The MIDS data contains data relating to pregnant women at their initial antenatal assessments, and mother and baby (or babies) data including intention to breastfeed at birth for all births. The NCCH comprises information pertaining to birth registration, monitoring of child health examinations, and immunisations. These records were linked at the individual level for all women known to be pregnant in Wales between 2018 and 2021.

Confidential: For Review Only

Data contained in the breastfeeding dataset included the outcome method. The outcomes are categorised in SAIL into exclusively breastfeeding, combined - predominantly breastfeeding, combined - partial breastfeeding, and artificial milk. The collection times were categorised into first feed, ten days, six weeks, and six months. They were asked whether they intended to breastfeed and only a yes or no response was noted. All missing (NA) outcome values were removed, and regression undertaken with the MIDS intention to breastfeed yes or no binary. Intention to feed from MIDS is compared to responses to 258 responses on the Born in Wales survey [13]. This survey asked expectant mothers how they were planning to feed their baby. Intention during pregnancy (Born in Wales) and after the birth (MIDS) was compared to see if responses were consistent. Informed consent was obtained by an information sheet and consent form provided to participants before starting the survey.

Patient and Public Involvement

Midwives from Swansea provided guidance and support during survey design and aided in recruitment by discussing the study at dating scans and routine appointments with expectant mothers.

Ethical Approval

The data held in the SAIL databank in Wales are anonymised. All data contained in SAIL has the permission from the relevant Caldicott Guardian or Data Protection Officer. This study is part of Born in Wales which was approved by the Health Research Authority on 16/06/2021, protocol number RIO 030-20.

Confidential: For Review Only

Statistical Analysis

Descriptive statistics were conducted on rates of breastfeeding per year from 2018 to 2021. Binary logistic regression models were utilised to examine the impact of the explanatory variables intention to breastfeed, age group and ethnicity on breastfeeding rates, reporting odds ratios (OR) with 95% confidence intervals and significance level accepted at $p<0.05$. For intention, 'yes'/'no' responses were coded as 1 and 0 respectively and that there was no other outcome to be coded. The reference groups were those aged <18, of white ethnicity, and those intending to breastfeed. The data handling and preparation for the descriptive statistics and logistic regression were performed in RStudio R Version 4.1.3 [14]. Final data preparation specific to these analyses such as setting the reference groups and descriptive statistics and logistic regression were performed in RStudio.

Confidential: For Review Only

Results

A total of 122,387 babies born to 106,022 mothers were identified from 2018 to 2021. Excluding those babies born outside of Wales (N=3,172), stillbirths (N=494) and those having no data available for breastfeeding outcome (N=1,721), the cohort for analysis of breastfeeding rates comprised of N=117,000 babies. For logistic regression, a further N=22,055 babies were excluded because of NA values for maternal age, intention to breastfeed and breastfeeding outcome. Resulting in the regression analysis being conducted on N=94,945 babies (Figure 1 describes the participants in the cohort). Most of the women were aged between 30-39 (46.7%) and 25-29 years (29.7%). The majority were White (81.9%) and intended to breastfeed (58.1%), see Table 1 for the demographics of the cohort. The study population (for regression) had higher numbers of women who did not intend to breastfeed (40%) compared to the Wales population database (33%).

Figure 1. Study flowchart explaining how the cohort was identified

		Full Wales Cohort		Study Population after exclusion	
**Category**		**N**	**%**	**N**	**%**
**Intention to breastfeed**	Yes	67956	58.1	56164	59.1
	No	38965	33.3	38781	40.9
	NA	10079	8.6	0	0

Confidential: For Review Only

**Maternal Age Group**	<18	933	0.8	816	0.9
	18-24	22711	19.4	19480	20.5
	25-29	34819	29.7	28737	30.3
	30-39	54588	46.7	42834	45.1
	40-50	3914	3.4	3069	3.2
	>50	10	0.0	10	0.0
	NA	25	0.0	0	0.0
**Ethnicity**	White	95842	81.9	78504	82.7
	Asian	3401	2.9	2614	2.7
	Black	1300	1.1	898	0.9
	Mixed	2426	2.1	1816	1.9
	Other	1169	1.0	809	0.9
	Unknown	12862	11.0	10304	10.9
	**Total**	**117,000**	**100**	**94,945**	**100**

145 *Table 1*. Demographics of the study cohort

Confidential: For Review Only

Rates of breastfeeding pre-pandemic, 2020 and 2021

Across the four years, between 59.9% and 61.9% of women breastfed their baby for their first feed. This decreased to between 35.1% and 37.3% breastfeeding at ten days, 25.4% to 28.5% at six weeks and those exclusively breastfeeding were lowest at six months at 16.6% to 20.6%. Between 11.7% and 14% at least partially breastfed at ten days, 7.5% to 8.4% at six weeks and 3.4% to 4.9% at six months. Alternative forms of feeding including a combination of breastmilk and artificial milk, and exclusively artificial milk were mostly adopted after the first feed. The biggest difference was breastfeeding to six months between pre-pandemic (16.6%) and in 2020 (20.6%) (difference: 3.99% (CI 3.35-4.62%)).

Examining the impact of intention to breastfeed, age, and ethnicity on breastfeeding outcome

The study found that the number of women intending to breastfeed did not differ during the pandemic (57.8% of expectant mothers intended to breastfeed before the pandemic compared to 58.7% in 2020 (difference: -0.88 (01.58,-0.18%))). Logistic regression was conducted to examine the variations in breastfeeding rates by intention to breastfeed, adjusted for age, year and ethnicity. The model explained 12% (R^2 Tjur) of the variance in exclusive breastfeeding. Those who intended to breastfeed were 27.47 times more likely to breastfeed for six months compared to those who did not intend to breastfeed (OR=27.47, 95% CI 24.79-30.54, p <0.001). Those aged 30-39 were 3.91 times more likely to breastfeed for six months compared to those aged <18 (OR=3.91, 95% CI 2.65-

Confidential: For Review Only

6.04, p < 0.001). Mothers in 2020 were 1.40 times more likely to breastfeed for six months compared to mothers pre-pandemic (OR=1.40, 95% CI 1.33-1.48, p < 0.001). Those of Black or Black British ethnic group were 1.88 times more likely to breastfeed for six months compared to White ethnic group (OR=1.88, CI 95% 1.60-2.20, p < 0.001), see Table 2 and Figure 2.

**Characteristic**		OR[1] (95% CI[2])
**Intention to breastfeed**	No	Reference
	Yes	27.47 (24.79-30.54)
**Maternal Age**	<18	Reference
	18-24	1.66 (1.12-2.57)
	25-29	2.76 (1.87-4.28)
	30-39	3.91 (2.65-6.04)
	40-50	3.74 (2.51-5.83)
**Ethnic Group**	White	Reference
	Asian	0.97 (0.87-1.08)
	Black	1.88 (1.60-2.20)
	Mixed	1.19 (1.05-1.35)
	Other	1.30 (1.08-1.55)
	Unknown	1.23 (1.16-1.31)

Confidential: For Review Only

**Year**	2018	Reference
	2019	1.08 (1.02-1.14)
	2020	1.40 (1.33-1.48)
	2021	0.91 (0.86-0.96)

Table 2. Binary Logistic Regression analysis of factors associated with breastfeeding outcome at six months. OR - Odds Ratio. CI - Confidence Interval.

Figure 2. Logistic regression for exclusive breastfeeding for six months

Intention to breastfeed in Born in Wales survey compared to MIDS data

Of 258 women who completed The Born in Wales survey during pregnancy and had a record in MIDS, 208 (80%) reported an intention to breastfeed. Of these, 88% (183/208) reported the intention to breastfeed again in the MIDS data (after the birth). 12% (25/208) had changed their mind and now intended to not breastfeed or were no longer sure. If a woman reported the intention to not breastfeed on the survey during pregnancy, they changed their mind and reported they would like to breastfeed in less than 9% of cases. This suggests among a survey population, the initial intention to breastfeed/not breastfeed only changes for about 10% of women.

Discussion

The findings indicate that approximately 58% of expectant mothers intend to breastfeed and across the four years approximately 60% of mothers in Wales breastfeed for their first feed. However, only 16.6% to 20.6% continue to exclusively breastfeed up to the WHO recommended duration of six months. The rates of breastfeeding to six months were highest during 2020 when the restrictions due to the COVID-19 pandemic were at its height although intention did not significantly change during 2020. This is in contrast to previous research that indicated COVID-19 negatively affected breastfeeding [9]. The rates of breastfeed to six months are now returning to pre pandemic levels. It is important to note that the change is unlikely to be just due to COVID-19. The pandemic did not occur in isolation and breastfeeding rates have been slowly increasing due to support initiatives.

A systematic review to identify factors influencing exclusive breastfeeding to six months has previously been conducted [15]. Exclusive breastfeeding up to six months was reportedly affected by maternal working status, breastfeeding knowledge, delivery mode, perception of insufficient human milk, mothers' infant feeding attitude, breastfeeding self-efficacy, and intention [15]. Therefore, breastfeeding up to six months is associated with employment status and breastfeeding knowledge. Supporting women to visualise how they might continue to breastfeed given their work commitments for example, where it is feasible to work from home, and improving knowledge early on in pregnancy could help to improve intention to breastfeed for longer.

Confidential: For Review Only

The timing of asking about intention to breastfeed is important to examine. If intention was asked at the start of the pregnancy and this led to a higher uptake of exclusive breastfeeding for six months, it would point to the need to influence women's perceptions of breastfeeding even prior to getting pregnant. For example, a social media campaign and advertising. It is rare to see adverts promoting breastfeeding outside of a healthcare setting but there are adverts for artificial milk. It would be interesting to have an in-depth study of women's reasons for and against breastfeeding, including the reasons why they stop breastfeeding and link these to the data.

Limitations

The study utilises healthcare data for pregnant women and new mothers in Wales including the maternity and child health data; thus providing a national perspective of breastfeeding rates established on a total population cohort, enabling the findings to be generalisable. However, it must be noted that it is difficult to fully know whether the data that was available on breastfeeding is reliable. The quality of data may be influenced by collection methods and how often health visitors were able to visit families to ask questions about methods of feeding and how long they breastfed. Use of expressed milk and donor milk were not explored in the current study and the datasets employed in this study did not contain information regarding donor milk or alternative methods. The survey with women will give a highly optimistic finding as the women who responded to a survey already had a higher rate of intention to breastfeed compared to the routine data population and so the findings from this survey will be biased toward breastfeeding.

Confidential: For Review Only

In conclusion, intention to breastfeed was the strongest predictor of exclusive breastfeeding outcome at six months. Therefore, more targeted interventions to promote, encourage and maintain breastfeeding duration up to six months need to be implemented early in pregnancy and in pregnancy planning.

Confidential: For Review Only

Author Contributions

All authors provided substantial contributions to the piece of work. HJ, MS, MM, and AB conducting the analysis and interpretation of the data. SB conception and design of the work. HJ, MJ, and NK drafting and revising the work critically for intellectual content. All authors contributed to the final approval of the version to be published. All authors agree to be accountable for all aspects of the work.

Conflict of interest statement

There are no conflicts of interest for any of the authors.

Funding Statement

This work is funded by the National Centre for Population Health and Wellbeing Research (NCPHWR) Grant Number: AMS103836 and the National Core Studies funded by the Medical Research Council (MRC) Grant Number: MC_PC_20030.

Confidential: For Review Only

References

[1] Pérez-Escamilla R. Breastfeeding in the 21st century: How we can make it work. *Soc. Sci. Med 2020*; 244,112331

[2] Victora CG, Bahl R, Barros AJ et al. Breastfeeding in the 21st century: epidemiology, mechanisms, and lifelong effect. *Lancet* 2016;387:475–490.

[3] Rollins NC, Bhandari N, Hajeebhoy N et al. Why invest, and what it will take to improve breastfeeding practices? *Lancet* 2016;387(10017):491-504.

[4] World Health Organization. Exclusive breastfeeding for six months best for babies everywhere. [WHO Website]. https://www.who.int/news/item/15-01-2011-exclusive-breastfeeding-for-six-months-best-for-babies-everywhere. Accessed December 13, 2022.

[5] Welsh Government. All Wales Breastfeeding Five Year Action Plan. [Welsh Government Website]. https://gov.wales/sites/default/files/publications/2019-06/all-wales-breastfeeding-five-year-action-plan-july-2019_0.pdf Accessed December 13, 2022.

[6] Hunt L, Thomson G, Whittaker K, Dykes F. Adapting breastfeeding support in areas of socio-economic deprivation: a case study approach. *Int. J. Equity Health* 2021;20:83.

[7] Meedya S, Fahy K, Kable A. Factors that positively influence breastfeeding duration to 6 months: a literature review. *Women Birth* 2010;23:135–145.

Confidential: For Review Only

[8] Milani GP, Porro A, Agostoni C, Giannì ML. Breastfeeding during a Pandemic. Ann. Nutr. Metab. 2022:1-9.

[9] Vazquez-Vazquez A, Dib S, Rougeaux E, Wells JC, Fewtrell MS. The impact of the Covid-19 lockdown on the experiences and feeding practices of new mothers in the UK: Preliminary data from the COVID-19 New Mum Study. *Appetite* 2021;156,104985.

[10] United Nations. Transforming our World: The 2030 Agenda for Sustainable Development. 2015. [United Nations Website]. https://sdgs.un.org/2030agenda . Accessed December 13, 2022.

[11] Jones KH, Ford DV, Thompson S, Lyons RA. A Profile of the SAIL Databank on the UK Secure Research Platform. *IJPDS* 2019;4(2).

[12] Lyons RA, Ford DV, Moore L, Rodgers SE. Use of data linkage to measure the population health effect of non-health-care interventions. *Lancet* 2014;383(9927):1517-1519.

[13] Born in Wales. Expectant Parents [Born in Wales Website]. 2022. https://ncphwr.org.uk/portfolio/born-in-wales-overview/ Accessed December 13, 2022.

[14] RStudio Team (2020). RStudio: Integrated Development for R. RStudio, PBC, Boston, MA URL http://www.rstudio.com/.

[15] Wu Q, Tang N, Wacharasin C. Factors influencing exclusive breastfeeding for 6 months postpartum: A systematic review. Int. J. Nurs. Knowl. 2022;33:4:290-303.

Confidential: For Review Only

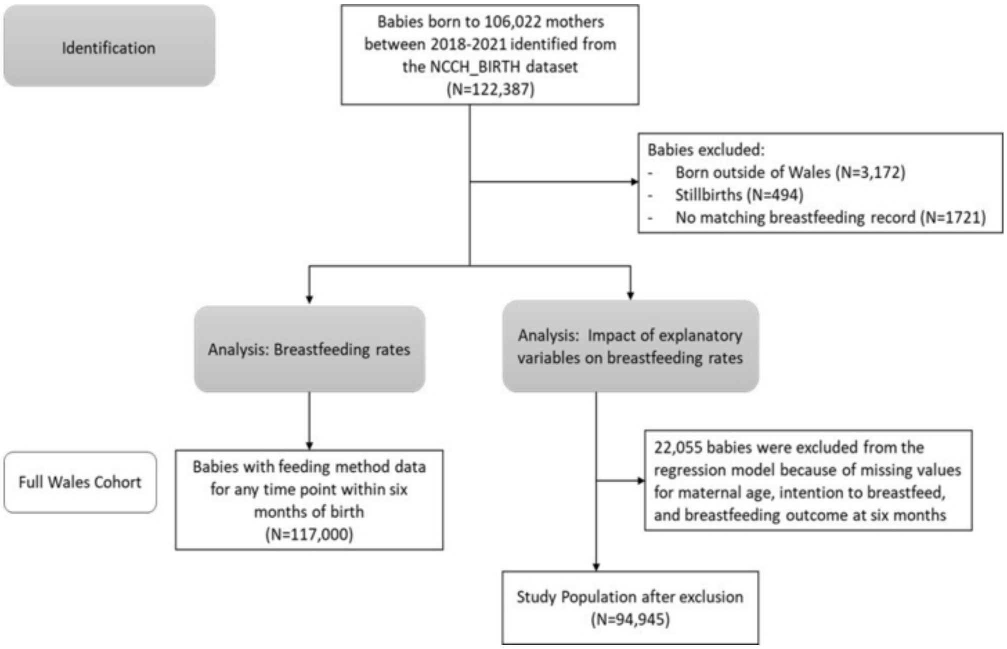

Figure 1. Study flowchart explaining how the cohort was identified

61x39mm (300 x 300 DPI)

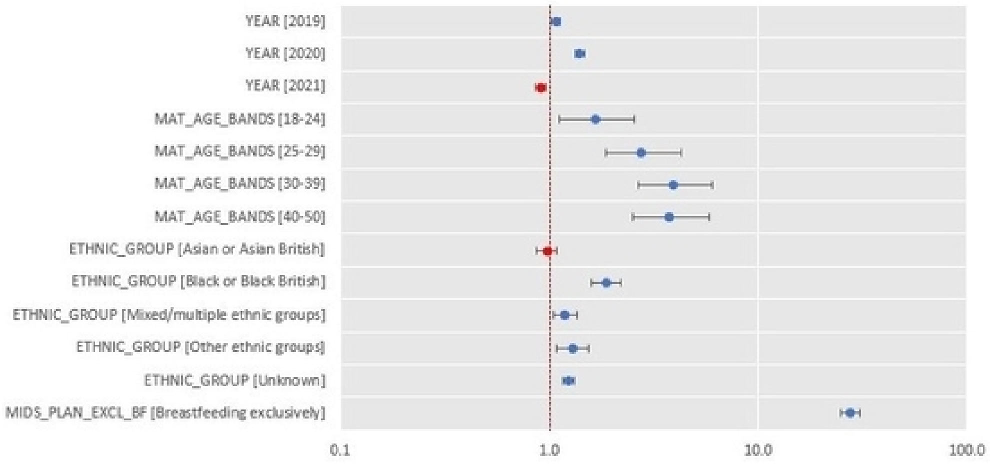

Figure 2. Logistic regression for exclusive breastfeeding for six months

47x22mm (300 x 300 DPI)

BMJ Paediatrics Open

Breastfeeding initiation and duration through the COVID-19 pandemic, a linked population-level routine data study - The Born in Wales cohort 2018-2021

Journal:	*BMJ Paediatrics Open*
Manuscript ID	bmjpo-2023-001907.R1
Article Type:	Original research
Date Submitted by the Author:	13-Mar-2023

Complete List of Authors:	Jones, Hope; Swansea University Medical School, National Centre for Population Health and Wellbeing Research, Faculty of Medicine, Health & Life Science<br>Seaborne, Mike; National Centre for Population Health and Wellbeing Research, Swansea University Medical School, Faculty of Medicine, Health & Life Science<br>Mhereeg, Mohamed; National Centre for Population Health and Wellbeing Research, Swansea University Medical School, Faculty of Medicine, Health & Life Science<br>James, Michaela; National Centre for Population Health and Wellbeing Research, Swansea University Medical School, Faculty of Medicine, Health & Life Science<br>Kennedy, Natasha; National Centre for Population Health and Wellbeing Research, Swansea University Medical School, Faculty of Medicine, Health & Life Science<br>Bandyopadhyay, Amrita; National Centre for Population Health and Wellbeing Research, Swansea University Medical School, Faculty of Medicine, Health & Life Science<br>Brophy, Sinead; National Centre for Population Health and Wellbeing Research, Swansea University Medical School, Faculty of Medicine, Health & Life Science

Keywords:	COVID-19, Data Collection

SCHOLARONE™
Manuscripts

I, the Submitting Author has the right to grant and does grant on behalf of all authors of the Work (as defined in the below author licence), an exclusive licence and/or a non-exclusive licence for contributions from authors who are: i) UK Crown employees; ii) where BMJ has agreed a CC-BY licence shall apply, and/or iii) in accordance with the terms applicable for US Federal Government officers or employees acting as part of their official duties; on a worldwide, perpetual, irrevocable, royalty-free basis to BMJ Publishing Group Ltd ("BMJ") its licensees and where the relevant Journal is co-owned by BMJ to the co-owners of the Journal, to publish the Work in this journal and any other BMJ products and to exploit all rights, as set out in our licence.

The Submitting Author accepts and understands that any supply made under these terms is made by BMJ to the Submitting Author unless you are acting as an employee on behalf of your employer or a postgraduate student of an affiliated institution which is paying any applicable article publishing charge ("APC") for Open Access articles. Where the Submitting Author wishes to make the Work available on an Open Access basis (and intends to pay the relevant APC), the terms of reuse of such Open Access shall be governed by a Creative Commons licence – details of these licences and which Creative Commons licence will apply to this Work are set out in our licence referred to above.

Other than as permitted in any relevant BMJ Author's Self Archiving Policies, I confirm this Work has not been accepted for publication elsewhere, is not being considered for publication elsewhere and does not duplicate material already published. I confirm all authors consent to publication of this Work and authorise the granting of this licence.

Confidential: For Review Only

Breastfeeding initiation and duration through the COVID-19 pandemic, a linked population-level routine data study - The Born in Wales cohort 2018-2021

Hope Jones[1], Mike Seaborne[1], Mohamed Mhereeg[1], Michaela James[1], Natasha Kennedy[1], Amrita Bandyopadhyay[1], Sinead Brophy[1]

Affiliations:

[1]National Centre for Population Health and Wellbeing Research, Swansea University Medical School, Faculty of Medicine, Health & Life Science, Swansea, Wales, UK.

Corresponding Author: h.e.jones@swansea.ac.uk, Hope Eleri Jones, Data Science Building, Swansea University, Swansea, Wales, SA2 8PP

Keywords: Breastfeeding, Intention, Exclusive Breastfeeding, Linked Data

Word Count: 2,495

Confidential: For Review Only

Abstract

Objective: The World Health Organisation recommends exclusive breastfeeding for the first six months of life. This study aimed to examine the impact the pandemic had on breastfeeding uptake and duration, and if intention to breastfeed is associated with longer duration of exclusive breastfeeding.

Methods: A cohort study utilising routinely collected, linked healthcare data from the Secure Anonymised Information Linkage databank (SAIL). All women that gave birth in Wales between 2018 and 2021 recorded in the Maternal Indicators (MIDS) dataset were asked about intention to breastfeed. This data was linked with the National Community Child Health (NCCH) Births and Breastfeeding dataset to examine breastfeeding rates.

Results: Intention to breastfeed was associated with being 27.6 times more likely to continue to exclusively breastfeed for six months compared to those who did not intend to breastfeed (OR 27.6, 95% CI: 24.9-30.7). Breastfeeding rates at six months were 16.6% pre-pandemic and 20.5% in 2020. When compared to a survey population, the initial intention to breastfeed/not breastfeed only changes for about 10% of women.

Conclusion: Women were more likely to exclusively breastfeed for six months during the pandemic compared to before or after the pandemic. Arguably, interventions which enable families to spend more time with their baby such as maternal and paternal leave may help improve breastfeeding duration. The biggest predictor of breastfeeding at six months was intention to breastfeed. Therefore, targeted interventions during pregnancy to encourage motivation to breastfeed could improve duration of breastfeeding.

Confidential: For Review Only

Key Messages

What is already known on this topic: The current World Health Organisation (WHO) recommendations indicate that mothers should provide exclusive breastfeeding to their babies during the first six months of life. However, the United Kingdom (UK) has one of the lowest breastfeeding rates in the world.

What this study adds: Intention to breastfeed was the strongest predictor of exclusive breastfeeding at six months

How this study might affect research, practice, or policy: Targeted interventions during pregnancy to encourage motivation to breastfeed could improve duration of breastfeeding

Confidential: For Review Only

Introduction

The health and social benefits of breastfeeding are already well-established [1]. Breastfeeding provides short-term and long-term physical, economic and environmental improvements for children, women, and society. Benefits to breastfed babies include fewer infections, increased intelligence, and protection against obesity and diabetes. Exclusively breastfed babies have been demonstrated to have higher intelligence quotient (IQ) [2]. They are also less prone to infections, asthma, and allergic diseases [3]. Moreover, breastfeeding is beneficial for mothers as research indicates it reduces the risk of breast, uterine and ovarian cancer, postpartum bleeding, and aids postpartum weight loss [4]. Breastfeeding supports short-term and long-lasting health environmental benefits for infants, mothers, and the wider population [5]. To recognise the advantages of breastfeeding, political support and financial investment are essential to protect, promote, and support breastfeeding [5].

The World Health Organisation (WHO) recommendations indicate that mothers should provide exclusive breastfeeding to their babies during the first six months of life [6]. However, the United Kingdom (UK) has one of the lowest breastfeeding rates in the world. 80% of babies are breastfed at birth but only 1% are exclusively breastfed for six months in the UK [7]. These breastfeeding rates are lower among women in areas of higher deprivation and health inequalities [8]. Breastfeeding rates in Wales have remained consistent despite investment in services and UNICEF baby friendly initiatives [7]. This is a multi-faceted issue relating to population health, initiation, and maintenance of breastfeeding. It is important to note that breastfeeding rates are

Confidential: For Review Only

influenced by various socio-economic factors, religion, education, and support services available [8].

Factors that influence women's breastfeeding decisions can include breastfeeding intention, self-efficacy and social support [9]. Exclusive breastfeeding intention describes a mother's intention to provide only breast milk for their baby. Self-efficacy refers to an individual's belief in their ability to complete the necessary behaviours to achieve specific outcomes. Breastfeeding promotion strategies conducted by midwives often include social support, however, there is limited research surrounding intention to breastfeed and self-efficacy and whether this impacts future breastfeeding behaviour.

A systematic review relating to intention to breastfeed has been conducted [10]. Characteristics associated with intention to breastfeed included first-time pregnancy, higher education level, older maternal age, prior breastfeeding experience, non-smoker, and living with a partner [10]. Further research has explored prenatal intention to breastfeed as a predictor of exclusive breastfeeding [11]. Intention to breastfeed was a strong predictor of breastfeeding behaviour, with a significant association with exclusive breastfeeding when leaving the hospital after birth [11]. However, this does not indicate whether exclusive breastfeeding continued up to six months.

In 2020, the COVID-19 pandemic and concerns about transmission of the disease contributed to higher rates of breastfeeding interruption [12]. Research found that the risk of transmission from infected mothers to their offspring is extremely low [12]. Available data showed that the lack of support for lactating mothers during the

Confidential: For Review Only

pandemic contributed to breastfeeding cessation worldwide [12]. Few strategies were proposed to overcome this issue. Research found that a third of women who planned to breastfeed reported that they did not receive help with positioning the infant [13]. Additionally, one in four women perceived that they did not get enough support with feeding in the hospital [13]. This could have been due to the increased burden on healthcare systems and pressure placed on healthcare professionals to discharge mothers sooner to minimise infection risks, resulting in fewer opportunities to support mothers with infant feeding.

The protection, promotion, and support of breastfeeding is a priority for public health. Therefore, encouraging optimal breastfeeding practices including breastfeeding initiation within one hour after birth, exclusive breastfeeding for six months, and continuation of breastfeeding for at least two years once nutritious and safe complementary foods are introduced at six months is crucial for meeting the Sustainable Development Goals by 2030 [14].

This study aimed to examine the impact the pandemic had on breastfeeding uptake and duration and if intention to breastfeed is associated with longer duration of exclusive breastfeeding. Understanding what factors impact exclusive breastfeeding to the recommended six months will help develop more targeted interventions to promote and support breastfeeding.

Confidential: For Review Only

Methods

Study Design and Setting

A cohort study utilising routinely collected anonymised population-level linked data from the Secure Anonymised Information Linkage (SAIL) databank [15,16]. Data sources include the Maternal Indicators (MIDS) and the National Community Child Health (NCCH) Births and Breastfeeding datasets. All women in Wales in the NCCH Breastfeeding and MIDS data were deemed eligible for analysis; any stillbirths were excluded. Breastfeeding rates were compared from 2018 to 2021.

Data Sources and Linkage

Analysis was undertaken using anonymised population-scale, individual level linked routinely collected data available in SAIL [15,16]. This anonymously links a wide range of person-based data using a unique, encrypted personal identifier. The linkage in this study includes the MIDS, NCCH Births and Breastfeeding data. MIDS contains data relating to pregnant women at their initial antenatal assessments, and mother and baby (or babies) data including intention to breastfeed at birth for all births. The NCCH comprises information pertaining to birth registration and monitoring of child health examinations. These records were linked for all women known to be pregnant in Wales between 2018 and 2021.

Data in the breastfeeding dataset included the outcome method. Outcomes are categorised in SAIL into exclusively breastfeeding, combined - predominantly breastfeeding, combined - partial breastfeeding, and artificial milk. For the purposes of

Confidential: For Review Only

the analyses in this study, the latter three categories were combined as ‘other feeds’ i.e., non-exclusive breastfeeding. The collection times were categorised into first feed, ten days, six weeks, and six months. Intention to breastfeed was recorded as yes or no Missing breastfeeding outcome and missing MIDS intention were categorised as unknown. Regression analysis was performed on the data. In order to examine how consistent the intention to breastfeed was we compared the responses from MIDS with responses on the Born in Wales survey [17]. This survey asked expectant mothers how they were planning to feed their baby. Intention during pregnancy (Born in Wales) and after the birth (MIDS) was compared. Informed consent was obtained by an information sheet and consent form provided to participants before starting the survey.

Patient and Public Involvement

Midwives from Swansea provided guidance and support during survey design and aided in recruitment by discussing the study at dating scans and routine appointments with expectant mothers.

Ethical Approval

The data held in the SAIL databank in Wales are anonymised. All data contained in SAIL has the permission from the relevant Caldicott Guardian or Data Protection Officer. This study is part of Born in Wales which was approved by the Health Research Authority on 16/06/2021, protocol number RIO 030-20.

Statistical Analysis

Confidential: For Review Only

Descriptive statistics were conducted on rates of breastfeeding per year from 2018 to 2021. Multinomial regression models were utilised to examine the impact of the explanatory variables intention to breastfeed (assessed on all women attending antenatal visits in Wales), age and ethnicity on breastfeeding rates, reporting odds ratios (OR) with 95% confidence intervals and significance level accepted at $p<0.05$. The reference groups were those of white ethnicity, and those not intending to breastfeed. All data wrangling and analyses with descriptive statistics and regression models were performed in RStudio R Version 4.1.3 [18].

Confidential: For Review Only

Results

122,389 babies born to 106,022 mothers were identified from 2018 to 2021. Excluding those babies born outside of Wales (N=3,172), stillbirths (N=494) and those having no matching breastfeeding dataset record (N=1,692), the cohort comprised N=117,031 babies. Figure 1 describes the participants in the cohort. The mean age of the cohort was 29.4 years. The majority were White (81.9%) and intended to breastfeed (58.1%). Table 1 describes the demographics of the cohort.

Figure 1. Study flowchart explaining how the cohort was identified

Category		N	%
**Intention to Breastfeed**	Yes	67,973	58.1
	No	38,975	33.3
	NA	10,083	8.6
**Maternal Age Group**	18-24	22,719	19.4
	25-29	34,831	29.7
	30-39	54,615	46.7
	40-50	3,917	3.3
	>50	10	0.0
	NA	1	0.0
**Ethnicity**	White	95,868	81.9
	Asian	3,402	2.9
	Black	1,300	1.1
	Mixed	2,427	2.1
	Other	1,169	1.0
	Unknown	12,865	11.0
**Total**		**117,031**	**100**

Table 1. Demographics of the study cohort

Confidential: For Review Only

Rates of breastfeeding pre-pandemic, 2020 and 2021

Across the four years, between 59.9% and 61.9% of women breastfed their baby for their first feed. This decreased to between 35.1% and 37.3% breastfeeding at ten days, 25.4% to 28.5% at six weeks and those exclusively breastfeeding were lowest at six months at 16.6% to 20.6% (See Figure 2). Between 11.7% and 14% at least partially breastfed at ten days, 7.5% to 8.4% at six weeks and 3.4% to 4.9% at six months. Alternative forms of feeding including a combination of breastmilk and artificial milk, and exclusively artificial milk were mostly adopted after the first feed. The biggest difference was breastfeeding to six months between pre-pandemic (16.6%) and in 2020 (20.6%) (difference: 3.99% (CI 3.35-4.62%)).

Figure 2. Exclusive breastfeeding of babies born in Wales between 2018-2021

Impact of intention to breastfeed, age, and ethnicity on breastfeeding outcome

The number of women intending to breastfeed did not differ during the pandemic (57.8% of expectant mothers intended to breastfeed before the pandemic compared to 58.7% in 2020 (difference: -0.88 (01.58,-0.18%))). Multinomial regression was conducted to examine the variations in breastfeeding rates by intention to breastfeed, adjusted for age, year and ethnicity. Those who intended to breastfeed were 27.6 times more likely to exclusively breastfeed for six months compared to those who did not intend to breastfeed (OR=27.6, 95% CI 24.9-30.7, $p < 0.001$). Mothers in 2020 were 1.41 times more likely to exclusively breastfeed for six months compared to mothers pre-pandemic (OR=1.41, 95% CI 1.34-1.48, $p < 0.001$). Those of Black or Black British ethnic group were 1.86 times more likely to exclusively breastfeed for six months

Confidential: For Review Only

compared to White ethnic group (OR=1.86, CI 95% 1.60-2.16, p < 0.001), see Table 2 and Figure 3.

Characteristic		OR[1] (95% CI[2])
Intention to Breastfeed	No	Reference
	Yes	27.6 (24.9-30.7)
	Unknown	15.7 (13.9-17.6)
Maternal Age	Continuous	1.06 (1,05-1.06)
Ethnic Group	White	Reference
	Asian	1.00 (0.90-1.11)
	Black	1.86 (1.60-2.16)
	Mixed	1.21 (1.07-1.36)
	Other	1.27 (1.07-1.51)
	Unknown	1.24 (1.18-1.31)
Year	2018	Reference
	2019	1.08 (1.02-1.13)
	2020	1.41 (1.34-1.48)
	2021	0.91 (0.86-0.96)

Table 2. Multinomial Regression analysis of factors associated with exclusive breastfeeding outcome at six months. OR - Odds Ratio. CI - Confidence Interval.

Figure 3. Multinomial regression for exclusive breastfeeding for six months

Born in Wales intention compared to MIDS intention

Of 258 women who completed The Born in Wales survey during pregnancy and had a record in MIDS, 208 (80%) reported an intention to breastfeed. Of these, 88% (183/208) reported the intention to breastfeed again in the MIDS data (after the birth). 12% (25/208) changed their mind and now intended to not breastfeed or were no longer sure. If a woman reported the intention to not breastfeed on the survey during pregnancy, they changed their mind and reported they would like to breastfeed in less than 9% of cases. This suggests among this survey population, the initial intention to breastfeed/not breastfeed only changed for about 10% of women.

Confidential: For Re

Discussion

The findings indicate that approximately 58% of expectant mothers intend to breastfeed and across the four years and approximately 60% of mothers in Wales breastfeed for their first feed. However, only 16.6% to 20.6% continue to exclusively breastfeed up to the WHO recommended duration of six months. The rates of breastfeeding to six months were highest during 2020 when the restrictions due to the COVID-19 pandemic were at its height although intention did not significantly change during 2020. This contrasts with previous research that indicated COVID-19 negatively affected breastfeeding [12]. All predictions were that breastfeeding rates would decline during the pandemic due to reduced access to health professional support, and lack of ability for direct social support from family and friends. However, this result suggests that despite the lack of external support options, more mothers were able to invest in exclusive breastfeeding. This might be explained by increased opportunities to remain at home. Having partners at home may have facilitated this. The immunological benefits of breastfeeding may also have contributed to some women pursuing exclusive breastfeeding for longer. However, the rates of exclusive breastfeeding to six months are now returning to pre pandemic levels.

A systematic review to identify factors influencing exclusive breastfeeding to six months has been conducted [19]. Exclusive breastfeeding to six months was reportedly affected by maternal working status, breastfeeding knowledge, delivery mode, perception of insufficient human milk, mothers' infant feeding attitude, exclusive breastfeeding self-efficacy, and intention [19]. Therefore, breastfeeding to six months is associated with working status and breastfeeding knowledge. Supporting women to visualise how they

Confidential: For Review Only

might continue to breastfeed given their work commitments for example, where it is feasible to work from home, and improving knowledge early on in pregnancy could help to improve intention to breastfeed for longer. The findings from Born in Wales may also suggest that time with a partner (e.g. as seen in COVID-19 lockdown) and so paternal leave may also help with breastfeeding duration. There are many reasons that breastfeeding may not be possible and so early intervention regarding problem solving and motivation to overcome barriers to breastfeed may facilitate sustained breastfeeding and improve intention to breastfeed. This is suggested by the strong association with intention to breastfeed and a longer duration of breastfeeding, indicating perhaps building motivation and problem solving early is the main facilitator to duration of breastfeeding.

The timing of asking about intention to breastfeed is important to examine. If intention was asked at the start of the pregnancy and this led to a higher uptake of exclusive breastfeeding for six months, it would point to the need to influence women's perceptions of breastfeeding even prior to getting pregnant. For example, a social media campaign and advertising. It is rare to see adverts promoting breastfeeding outside of a healthcare setting but there are adverts for artificial milk. The Lancet series on breastfeeding discusses inappropriate marketing tactics of artificial milk companies [20]. The series stresses that sales of artificial milk are directed by well-resourced marketing strategies that represent artificial milk with limited evidence, as recommendations for child health and developmental issues in a manner that undermines breastfeeding [20]. To build an environment for breastfeeding that is not tainted by marketing influences would require stronger political commitment, financial investment and high-level support.

Confidential: For Review Only

Further investigation would uncover more about the strength of intention and what women understand when asked about their intention to breastfeed. This would clarify whether stating that they intend to breastfeed means they intend to breastfeed immediately after birth, only while in hospital or exclusively for the recommended six months.

Limitations

The study utilises healthcare data for pregnant women and new mothers in Wales including maternity and child health data; thus providing a national perspective of breastfeeding rates, enabling the findings to be generalisable. However, it is difficult to know whether the data that was available on breastfeeding is reliable. The quality of data may be influenced by collection methods and how often health visitors were able to visit families to ask questions about methods of feeding and how long they breastfed. Use of expressed milk and donor milk were not explored in the current study and the datasets employed in this study did not contain information regarding alternative methods. The survey will give a highly optimistic finding as the women who responded to a survey already had higher intention to breastfeed compared to the routine data population so the findings from this survey will be biased toward breastfeeding.

In conclusion, aspects of the pandemic such as working from home or time with their partner may have improved breastfeeding duration and so policies and practices that facilitate time with the family may improve duration of breastfeeding. The initiation of breastfeeding was not influenced by the pandemic so protection from infection did not appear to encourage more women to start breastfeeding. Intention to breastfeed was the

Confidential: For Review Only

strongest predictor of exclusive breastfeeding at six months and therefore intervention early in the pregnancy or before pregnancy which facilitates motivation to breastfeed may improve breastfeeding duration.

Confidential: For Review Only

Author Contributions

All authors provided substantial contributions to the piece of work. HJ, MS, MM, and AB conducting the analysis and interpretation of the data. SB conception and design of the work. HJ, MJ, and NK drafting and revising the work critically for intellectual content. All authors contributed to the final approval of the version to be published. All authors agree to be accountable for all aspects of the work.

Conflict of interest statement

There are no conflicts of interest for any of the authors.

Funding Statement

This work is funded by the National Centre for Population Health and Wellbeing Research (NCPHWR) Grant Number: AMS103836 and the National Core Studies funded by the Medical Research Council (MRC) Grant Number: MC_PC_20030.

Confidential: For Review Only

References

[1] Pérez-Escamilla R. Breastfeeding in the 21st century: How we can make it work. *Soc. Sci. Med 2020*; 244,112331

[2] Feldman-Winter L. Where We Stand: Breastfeeding [Internet]. HealthyChildren.org. 2020. Available from: https://www.healthychildren.org/English/ages-stages/baby/breastfeeding/Pages/Where-We-Stand-Breastfeeding.aspx

[3] Nuzzi G, Di Cicco ME, Peroni DG. Breastfeeding and Allergic Diseases: What's New? Children (Basel). 2021;8(5):330. doi: 10.3390/children8050330. PMID: 33923294; PMCID: PMC8145659.

[4] Victora CG, Bahl R, Barros AJ et al. Breastfeeding in the 21st century: epidemiology, mechanisms, and lifelong effect. *Lancet* 2016;387:475–490.

[5] Rollins NC, Bhandari N, Hajeebhoy N et al. Why invest, and what it will take to improve breastfeeding practices? *Lancet* 2016;387(10017):491-504.

[6] World Health Organization. Exclusive breastfeeding for six months best for babies everywhere. [WHO Website]. https://www.who.int/news/item/15-01-2011-exclusive-breastfeeding-for-six-months-best-for-babies-everywhere. Accessed December 13, 2022.

[7] Welsh Government. All Wales Breastfeeding Five Year Action Plan. [Welsh Government Website]. https://gov.wales/sites/default/files/publications/2019-06/all-

Confidential: For Review Only

wales-breastfeeding-five-year-action-plan-july-2019_0.pdf Accessed December 13, 2022.

[8] Hunt L, Thomson G, Whittaker K, Dykes F. Adapting breastfeeding support in areas of socio-economic deprivation: a case study approach. *Int. J. Equity Health* 2021;20:83.

[9] Meedya S, Fahy K, Kable A. Factors that positively influence breastfeeding duration to 6 months: a literature review. *Women Birth* 2010;23:135–145.

[10] Vieira TO, Martins CD, Santana GS, Vieira GO, Silva LR. Intenção materna de amamentar: revisão sistemática [Maternal intention to breastfeed: a systematic review]. Cien Saude Colet. 2016;21(12):3845-3858. Portuguese. doi: 10.1590/1413-812320152112.17962015. PMID: 27925124.

[11] Linares AM, Rayens MK, Gomez ML. et al. Intention to Breastfeed as a Predictor of Initiation of Exclusive Breastfeeding in Hispanic Women. J Immigrant Minority Health. 2015;17, 1192–1198. doi.org/10.1007/s10903-014-0049-0

[12] Milani GP, Porro A, Agostoni C, Giannì ML. Breastfeeding during a Pandemic. Ann. Nutr. Metab. 2022:1-9.

[13] Vazquez-Vazquez A, Dib S, Rougeaux E, Wells JC, Fewtrell MS. The impact of the Covid-19 lockdown on the experiences and feeding practices of new mothers in the UK: Preliminary data from the COVID-19 New Mum Study. *Appetite* 2021;156,104985.

Confidential: For Review Only

294 [14] United Nations. Transforming our World: The 2030 Agenda for Sustainable
295 Development. 2015. [United Nations Website]. https://sdgs.un.org/2030agenda .
296 Accessed December 13, 2022.

297 [15] Jones KH, Ford DV, Thompson S, Lyons RA. A Profile of the SAIL Databank on the
298 UK Secure Research Platform. *IJPDS* 2019;4(2).

299 [16] Lyons RA, Ford DV, Moore L, Rodgers SE. Use of data linkage to measure the
300 population health effect of non-health-care interventions. *Lancet* 2014;383(9927):1517-
301 1519.

302 [17] Born in Wales. Expectant Parents [Born in Wales Website]. 2022.
303 https://ncphwr.org.uk/portfolio/born-in-wales-overview/ Accessed December 13, 2022.

304 [18] RStudio Team (2020). RStudio: Integrated Development for R. RStudio, PBC,
305 Boston, MA URL http://www.rstudio.com/.

306 [19] Wu Q, Tang N, Wacharasin C. Factors influencing exclusive breastfeeding for 6
307 months postpartum: A systematic review. Int. J. Nurs. Knowl. 2022;33:4:290-303.

308 [20] Rollins N, Piwoz E, Baker P, Kingston G, Mabaso KM, McCoy D. Marketing of
309 commercial milk formula: a system to capture parents, communities, science, and
310 policy. Lancet. 2023;401:10375.486-502 DOI:https://doi.org/10.1016/S0140-
311 6736(22)01931-6

312

Confidential: For Review Only

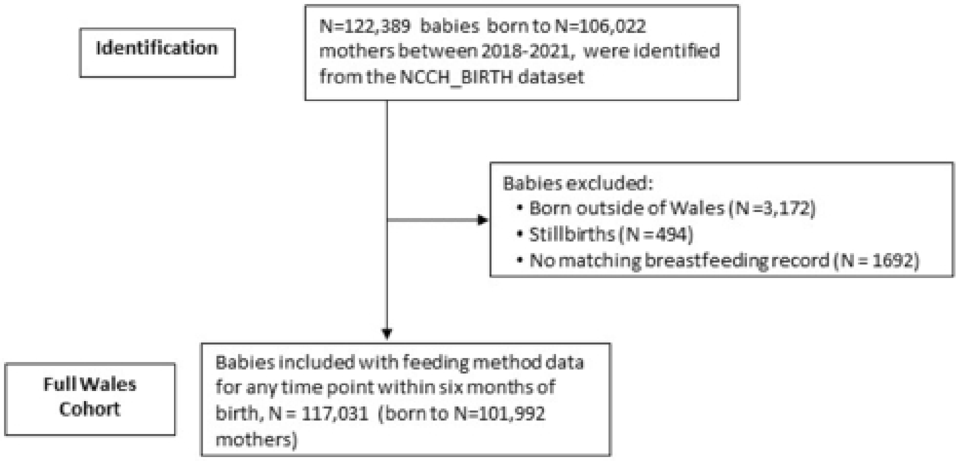

Figure 1. Study flowchart explaining how the cohort was identified

43x20mm (300 x 300 DPI)

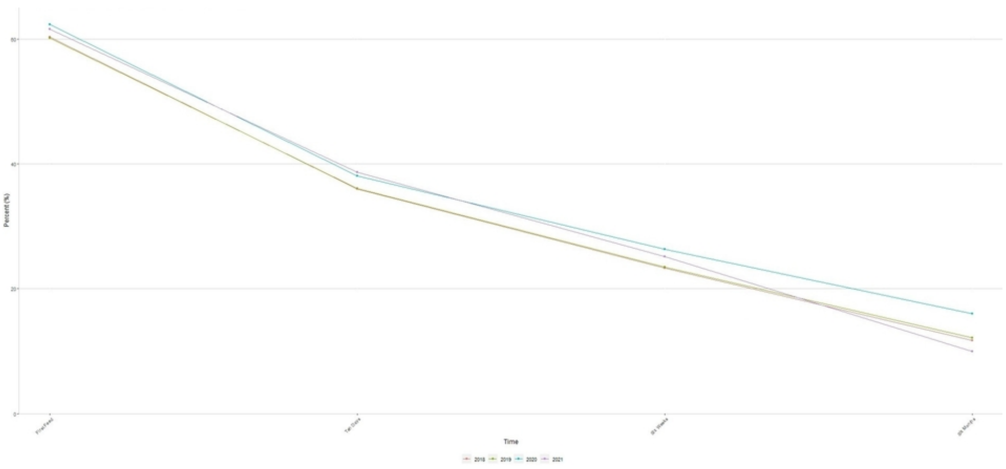

Figure 2. Exclusive breastfeeding of babies born in Wales between 2018-2021

80x36mm (300 x 300 DPI)

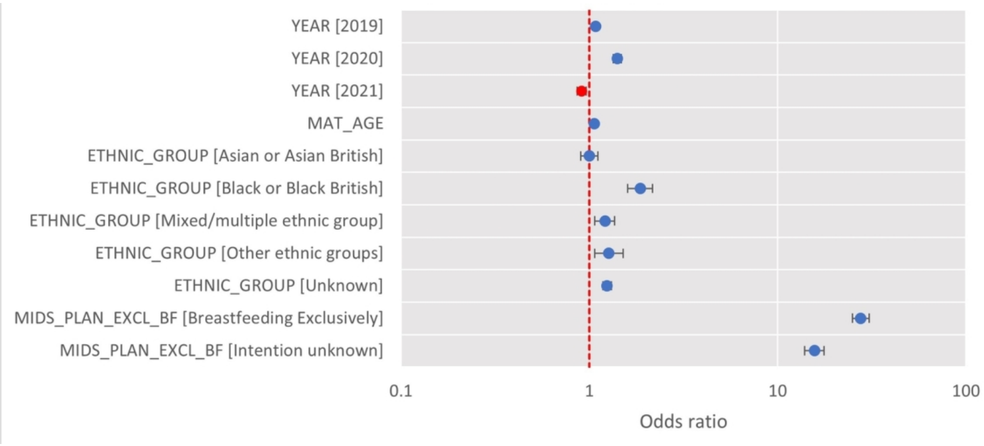

Figure 3. Multinomial regression for exclusive breastfeeding for six months

140x61mm (300 x 300 DPI)

BMJ Paediatrics Open

Breastfeeding initiation and duration through the COVID-19 pandemic, a linked population-level routine data study - The Born in Wales cohort 2018-2021

Journal:	*BMJ Paediatrics Open*
Manuscript ID	bmjpo-2023-001907.R2
Article Type:	Original research
Date Submitted by the Author:	18-Apr-2023
Complete List of Authors:	Jones, Hope; Swansea University Medical School, National Centre for Population Health and Wellbeing Research, Faculty of Medicine, Health & Life Science<br>Seaborne, Mike; National Centre for Population Health and Wellbeing Research, Swansea University Medical School, Faculty of Medicine, Health & Life Science<br>Mhereeg, Mohamed; National Centre for Population Health and Wellbeing Research, Swansea University Medical School, Faculty of Medicine, Health & Life Science<br>James, Michaela; National Centre for Population Health and Wellbeing Research, Swansea University Medical School, Faculty of Medicine, Health & Life Science<br>Kennedy, Natasha; National Centre for Population Health and Wellbeing Research, Swansea University Medical School, Faculty of Medicine, Health & Life Science<br>Bandyopadhyay, Amrita; National Centre for Population Health and Wellbeing Research, Swansea University Medical School, Faculty of Medicine, Health & Life Science<br>Brophy, Sinead; National Centre for Population Health and Wellbeing Research, Swansea University Medical School, Faculty of Medicine, Health & Life Science
Keywords:	COVID-19, Data Collection

SCHOLARONE™
Manuscripts

I, the Submitting Author has the right to grant and does grant on behalf of all authors of the Work (as defined in the below author licence), an exclusive licence and/or a non-exclusive licence for contributions from authors who are: i) UK Crown employees; ii) where BMJ has agreed a CC-BY licence shall apply, and/or iii) in accordance with the terms applicable for US Federal Government officers or employees acting as part of their official duties; on a worldwide, perpetual, irrevocable, royalty-free basis to BMJ Publishing Group Ltd ("BMJ") its licensees and where the relevant Journal is co-owned by BMJ to the co-owners of the Journal, to publish the Work in this journal and any other BMJ products and to exploit all rights, as set out in our licence.

The Submitting Author accepts and understands that any supply made under these terms is made by BMJ to the Submitting Author unless you are acting as an employee on behalf of your employer or a postgraduate student of an affiliated institution which is paying any applicable article publishing charge ("APC") for Open Access articles. Where the Submitting Author wishes to make the Work available on an Open Access basis (and intends to pay the relevant APC), the terms of reuse of such Open Access shall be governed by a Creative Commons licence – details of these licences and which Creative Commons licence will apply to this Work are set out in our licence referred to above.

Other than as permitted in any relevant BMJ Author's Self Archiving Policies, I confirm this Work has not been accepted for publication elsewhere, is not being considered for publication elsewhere and does not duplicate material already published. I confirm all authors consent to publication of this Work and authorise the granting of this licence.

Confidential: For Review Only

Breastfeeding initiation and duration through the COVID-19 pandemic, a linked population-level routine data study - The Born in Wales cohort 2018-2021

Hope Jones[1], Mike Seaborne[1], Mohamed Mhereeg[1], Michaela James[1], Natasha Kennedy[1], Amrita Bandyopadhyay[1], Sinead Brophy[1]

Affiliations:

[1]National Centre for Population Health and Wellbeing Research, Swansea University Medical School, Faculty of Medicine, Health & Life Science, Swansea, Wales, UK.

Corresponding Author: h.e.jones@swansea.ac.uk, Hope Eleri Jones, Data Science Building, Swansea University, Swansea, Wales, SA2 8PP

Keywords: Breastfeeding, Intention, Exclusive Breastfeeding, Linked Data

Word Count: 2,495

Confidential: For Review Only

Abstract

Objective: The World Health Organisation recommends exclusive breastfeeding for the first six months of life. This study aimed to examine the impact the pandemic had on breastfeeding uptake and duration, and whether intention to breastfeed is associated with longer duration of exclusive breastfeeding.

Methods: A cohort study utilising routinely collected, linked healthcare data from the Secure Anonymised Information Linkage databank (SAIL). All women that gave birth in Wales between 2018 and 2021 recorded in the Maternal Indicators (MIDS) dataset were asked about intention to breastfeed. This data was linked with the National Community Child Health (NCCH) Births and Breastfeeding dataset to examine breastfeeding rates.

Results: Intention to breastfeed was associated with being 27.6 times more likely to continue to exclusively breastfeed for six months compared to those who did not intend to breastfeed (OR 27.6, 95% CI: 24.9-30.7). Breastfeeding rates at six months were 16.6% pre-pandemic and 20.5% in 2020. When compared to a survey population, the initial intention to breastfeed/not breastfeed only changes for about 10% of women.

Conclusion: Women were more likely to exclusively breastfeed for six months during the pandemic compared to before or after the pandemic. Arguably, interventions which enable families to spend more time with their baby such as maternal and paternal leave may help improve breastfeeding duration. The biggest predictor of breastfeeding at six months was intention to breastfeed. Therefore, targeted interventions during pregnancy to encourage motivation to breastfeed could improve duration of breastfeeding.

Confidential: For Review Only

Key Messages

What is already known on this topic: The current World Health Organisation (WHO) recommendations indicate that mothers should provide exclusive breastfeeding to their babies during the first six months of life. However, the United Kingdom (UK) has one of the lowest breastfeeding rates in the world.

What this study adds: Breastfeeding rates at 6 months were significantly higher during the COVID-19 pandemic. Black mothers were significantly more likely to exclusively breastfeed for 6 months. Intention to breastfeed was the strongest predictor of exclusive breastfeeding at six months.

How this study might affect research, practice, or policy: Targeted interventions during pregnancy to encourage motivation to breastfeed could improve duration of breastfeeding.

Confidential: For Review Only

Introduction

The health and social benefits of breastfeeding are already well-established [1]. Breastfeeding provides short-term and long-term physical, economic and environmental improvements for children, women, and society. Benefits to breastfed babies include fewer infections, increased intelligence, and protection against obesity and diabetes. Exclusively breastfed babies have been demonstrated to have higher intelligence quotient (IQ) [2]. They are also less prone to infections, asthma, and allergic diseases [3]. Moreover, breastfeeding is beneficial for mothers as research indicates it reduces the risk of breast, uterine and ovarian cancer, postpartum bleeding, and aids postpartum weight loss [4]. Breastfeeding supports short-term and long-lasting health environmental benefits for infants, mothers, and the wider population [5]. To recognise the advantages of breastfeeding, political support and financial investment are essential to protect, promote, and support breastfeeding [5].

The World Health Organisation (WHO) recommendations indicate that mothers should provide exclusive breastfeeding to their babies during the first six months of life [6]. However, the United Kingdom (UK) has one of the lowest breastfeeding rates in the world. 80% of babies are breastfed at birth but only 1% are exclusively breastfed for six months in the UK [7]. These breastfeeding rates are lower among women in areas of higher deprivation and health inequalities [8]. Breastfeeding rates in Wales have remained consistent despite investment in services and UNICEF baby friendly initiatives [7]. This is a multi-faceted issue relating to population health, initiation, and maintenance of breastfeeding. It is important to note that breastfeeding rates are

Confidential: For Review Only

influenced by various socio-economic factors, religion, education, and support services available [8].

Factors that influence women's breastfeeding decisions can include breastfeeding intention, self-efficacy and social support [9]. Exclusive breastfeeding intention describes a mother's intention to provide only breast milk for their baby. Self-efficacy refers to an individual's belief in their ability to complete the necessary behaviours to achieve specific outcomes. Breastfeeding promotion strategies conducted by midwives often include social support, however, there is limited research surrounding intention to breastfeed and self-efficacy and whether this impacts future breastfeeding behaviour.

A systematic review relating to intention to breastfeed has been conducted [10]. Characteristics associated with intention to breastfeed included first-time pregnancy, higher education level, older maternal age, prior breastfeeding experience, non-smoker, and living with a partner [10]. Further research has explored prenatal intention to breastfeed as a predictor of exclusive breastfeeding [11]. Intention to breastfeed was a strong predictor of breastfeeding behaviour, with a significant association with exclusive breastfeeding when leaving the hospital after birth [11]. However, this does not indicate whether exclusive breastfeeding continued up to six months.

In 2020, the COVID-19 pandemic and concerns about transmission of the disease contributed to higher rates of breastfeeding interruption [12]. Research found that the risk of transmission from infected mothers to their offspring is extremely low [12]. Available data showed that the lack of support for lactating mothers during the

Confidential: For Review Only

pandemic contributed to breastfeeding cessation worldwide [12]. Few strategies were proposed to overcome this issue. Research found that a third of women who planned to breastfeed reported that they did not receive help with positioning the infant [13]. Additionally, one in four women perceived that they did not get enough support with feeding in the hospital [13]. This could have been due to the increased burden on healthcare systems and pressure placed on healthcare professionals to discharge mothers sooner to minimise infection risks, resulting in fewer opportunities to support mothers with infant feeding.

The protection, promotion, and support of breastfeeding is a priority for public health. Therefore, encouraging optimal breastfeeding practices including breastfeeding initiation within one hour after birth, exclusive breastfeeding for six months, and continuation of breastfeeding for at least two years once nutritious and safe complementary foods are introduced at six months is crucial for meeting the Sustainable Development Goals by 2030 [14].

This study aimed to examine the impact the pandemic had on breastfeeding uptake and duration and whether intention to breastfeed is associated with longer duration of exclusive breastfeeding. Understanding what factors impact exclusive breastfeeding to the recommended six months will help develop more targeted interventions to promote and support breastfeeding.

Confidential: For Review Only

Methods

Study Design and Setting

A cohort study utilising routinely collected anonymised population-level linked data from the Secure Anonymised Information Linkage (SAIL) databank [15,16]. Data sources include the Maternal Indicators (MIDS) and the National Community Child Health (NCCH) Births and Breastfeeding datasets. All women in Wales in the NCCH Breastfeeding and MIDS data were deemed eligible for analysis; any stillbirths were excluded. Breastfeeding rates were compared from 2018 to 2021.

Data Sources and Linkage

Analysis was undertaken using anonymised population-scale, individual level linked routinely collected data available in SAIL [15,16]. This anonymously links a wide range of person-based data using a unique, encrypted personal identifier. The linkage in this study includes the MIDS, NCCH Births and Breastfeeding data. MIDS contains data relating to pregnant women at their initial antenatal assessments, and mother and baby (or babies) data including intention to breastfeed at birth for all births. The NCCH comprises information pertaining to birth registration and monitoring of child health examinations. These records were linked for all women known to be pregnant in Wales between 2018 and 2021.

Data in the breastfeeding dataset included the outcome method. Outcomes are categorised in SAIL into exclusively breastfeeding, combined - predominantly breastfeeding, combined - partial breastfeeding, and artificial milk. For the purposes of

Confidential: For Review Only

the analyses in this study, the latter three categories were combined as ‘other feeds’ i.e., non-exclusive breastfeeding. The collection times were categorised into first feed, ten days, six weeks, and six months. Intention to breastfeed was recorded as yes or no Missing breastfeeding outcome and missing MIDS intention were categorised as unknown. Regression analysis was performed on the data. In order to examine how consistent the intention to breastfeed was we compared the responses from MIDS with responses on the Born in Wales survey [17]. This survey asked expectant mothers how they were planning to feed their baby. Intention during pregnancy (Born in Wales) and after the birth (MIDS) was compared. Informed consent was obtained by an information sheet and consent form provided to participants before starting the survey.

Patient and Public Involvement

Midwives from Swansea provided guidance and support during survey design and aided in recruitment by discussing the study at dating scans and routine appointments with expectant mothers.

Ethical Approval

The data held in the SAIL databank in Wales are anonymised. All data contained in SAIL has the permission from the relevant Caldicott Guardian or Data Protection Officer. This study is part of Born in Wales which was approved by the Health Research Authority on 16/06/2021, protocol number RIO 030-20.

Statistical Analysis

Confidential: For Review Only

Descriptive statistics were conducted on rates of breastfeeding per year from 2018 to 2021. Multinomial regression models were utilised to examine the impact of the explanatory variables intention to breastfeed (assessed on all women attending antenatal visits in Wales), age and ethnicity on breastfeeding rates, reporting odds ratios (OR) with 95% confidence intervals and significance level accepted at $p<0.05$. The reference groups were those of white ethnicity, and those not intending to breastfeed. All data cleaning and analyses with descriptive statistics and regression models were performed in RStudio R Version 4.1.3 [18].

Confidential: For Review Only

Results

122,389 babies born to 106,022 mothers were identified from 2018 to 2021. Excluding those babies born outside of Wales (N=3,172), stillbirths (N=494) and those having no matching breastfeeding dataset record (N=1,692), the cohort comprised N=117,031 babies. Figure 1 describes the participants in the cohort. The mean age of the cohort was 29.4 years. The majority were White (81.9%) and intended to breastfeed (58.1%). Table 1 describes the demographics of the cohort.

Figure 1. Study flowchart explaining how the cohort was identified

Category		N	%
**Intention to Breastfeed**	Yes	67,973	58.1
	No	38,975	33.3
	NA	10,083	8.6
**Maternal Age Group**	18-24	22,719	19.4
	25-29	34,831	29.7
	30-39	54,615	46.7
	40-50	3,917	3.3
	>50	10	0.0
	NA	1	0.0
**Ethnicity**	White	95,868	81.9
	Asian	3,402	2.9
	Black	1,300	1.1
	Mixed	2,427	2.1
	Other	1,169	1.0
	Unknown	12,865	11.0
**Total**		**117,031**	**100**

Table 1. Demographics of the study cohort

Confidential: For Review Only

Rates of breastfeeding pre-pandemic, 2020 and 2021

Across the four years, between 59.9% and 61.9% of women breastfed their baby for their first feed. This decreased to between 35.1% and 37.3% breastfeeding at ten days, 25.4% to 28.5% at six weeks and those exclusively breastfeeding were lowest at six months at 16.6% to 20.6% (See Figure 2). Between 11.7% and 14% at least partially breastfed at ten days, 7.5% to 8.4% at six weeks and 3.4% to 4.9% at six months. Alternative forms of feeding including a combination of breastmilk and artificial milk, and exclusively artificial milk were mostly adopted after the first feed. The biggest difference was breastfeeding to six months between pre-pandemic (16.6%) and in 2020 (20.6%) (difference: 3.99% (CI 3.35-4.62%)).

Figure 2. Exclusive breastfeeding of babies born in Wales between 2018-2021

Impact of intention to breastfeed, age, and ethnicity on breastfeeding outcome

The number of women intending to breastfeed did not differ during the pandemic (57.8% of expectant mothers intended to breastfeed before the pandemic compared to 58.7% in 2020 (difference: -0.88 (01.58,-0.18%))). Multinomial regression was conducted to examine the variations in breastfeeding rates by intention to breastfeed, adjusted for age, year and ethnicity. Those who intended to breastfeed were 27.6 times more likely to exclusively breastfeed for six months compared to those who did not intend to breastfeed (OR=27.6, 95% CI 24.9-30.7, $p < 0.001$). Mothers in 2020 were 1.41 times more likely to exclusively breastfeed for six months compared to mothers pre-pandemic (OR=1.41, 95% CI 1.34-1.48, $p < 0.001$). Those of Black or Black British ethnic group were 1.86 times more likely to exclusively breastfeed for six months

Confidential: For Review Only

compared to White ethnic group (OR=1.86, CI 95% 1.60-2.16, $p < 0.001$), see Table 2 and Figure 3.

Characteristic		OR[1] (95% CI[2])
**Intention to Breastfeed**	No	Reference
	Yes	27.6 (24.9-30.7)
	Unknown	15.7 (13.9-17.6)
**Maternal Age**	Continuous	1.06 (1,05-1.06)
**Ethnic Group**	White	Reference
	Asian	1.00 (0.90-1.11)
	Black	1.86 (1.60-2.16)
	Mixed	1.21 (1.07-1.36)
	Other	1.27 (1.07-1.51)
	Unknown	1.24 (1.18-1.31)
**Year**	2018	Reference
	2019	1.08 (1.02-1.13)
	2020	1.41 (1.34-1.48)
	2021	0.91 (0.86-0.96)

Table 2. Multinomial Regression analysis of factors associated with exclusive breastfeeding outcome at six months. OR - Odds Ratio. CI - Confidence Interval.

Figure 3. Multinomial regression for exclusive breastfeeding for six months

Confidential: For Re

Born in Wales intention compared to MIDS intention

Of 258 women who completed The Born in Wales survey during pregnancy and had a record in MIDS, 208 (80%) reported an intention to breastfeed. Of these, 88% (183/208) reported the intention to breastfeed again in the MIDS data (after the birth). 12% (25/208) changed their mind and now intended to not breastfeed or were no longer sure. When a woman reported the intention to not breastfeed on the survey during pregnancy, they changed their mind and reported they would like to breastfeed in less than 9% of cases. This suggests among this survey population, the initial intention to breastfeed/not breastfeed only changed for about 10% of women.

Discussion

The findings indicate that approximately 58% of expectant mothers intend to breastfeed and across the four years and approximately 60% of mothers in Wales breastfeed for their first feed. However, only 16.6% to 20.6% continue to exclusively breastfeed up to the WHO recommended duration of six months. The rates of breastfeeding to six months were highest during 2020 when the restrictions due to the COVID-19 pandemic were at its height although intention did not significantly change during 2020. This contrasts with previous research that indicated COVID-19 negatively affected breastfeeding [12]. All predictions were that breastfeeding rates would decline during the pandemic due to reduced access to health professional support, and lack of ability for direct social support from family and friends. However, this result suggests that despite the lack of external support options, more mothers were able to invest in exclusive breastfeeding. This might be explained by increased opportunities to remain at home. Having partners at home may have facilitated this. The immunological benefits of breastfeeding may also have contributed to some women pursuing exclusive breastfeeding for longer. However, the rates of exclusive breastfeeding to six months are now returning to pre pandemic levels.

A systematic review to identify factors influencing exclusive breastfeeding to six months has been conducted [19]. Exclusive breastfeeding to six months was reportedly affected by maternal working status, breastfeeding knowledge, delivery mode, perception of insufficient human milk, mothers' infant feeding attitude, exclusive breastfeeding self-efficacy, and intention [19]. Therefore, breastfeeding to six months is associated with working status and breastfeeding knowledge. Supporting women to visualise how they

Confidential: For Review Only

might continue to breastfeed given their work commitments for example, where it is feasible to work from home, and improving knowledge early on in pregnancy could help to improve intention to breastfeed for longer. The findings from Born in Wales may also suggest that time with a partner (e.g. as seen in COVID-19 lockdown) and so paternal leave may also help with breastfeeding duration. There are many reasons that breastfeeding may not be possible and so early intervention regarding problem solving and motivation to overcome barriers to breastfeed may facilitate sustained breastfeeding and improve intention to breastfeed. This is suggested by the strong association with intention to breastfeed and a longer duration of breastfeeding, indicating perhaps building motivation and problem solving early is the main facilitator to duration of breastfeeding.

The timing of asking about intention to breastfeed is important to examine. If intention was asked at the start of the pregnancy and this led to a higher uptake of exclusive breastfeeding for six months, it would point to the need to influence women's perceptions of breastfeeding even prior to getting pregnant. For example, a social media campaign and advertising. It is rare to see adverts promoting breastfeeding outside of a healthcare setting but there are adverts for artificial milk. The Lancet series on breastfeeding discusses inappropriate marketing tactics of artificial milk companies [20]. The series stresses that sales of artificial milk are directed by well-resourced marketing strategies that represent artificial milk with limited evidence, as recommendations for child health and developmental issues in a manner that undermines breastfeeding [20]. To build an environment for breastfeeding that is not tainted by marketing influences would require stronger political commitment, financial investment and high-level support.

Confidential: For Review Only

Further investigation would uncover more about the strength of intention and what women understand when asked about their intention to breastfeed. This would clarify whether stating that they intend to breastfeed means they intend to breastfeed immediately after birth, only while in hospital or exclusively for the recommended six months.

Limitations

The study utilises healthcare data for pregnant women and new mothers in Wales including maternity and child health data; thus providing a national perspective of breastfeeding rates, enabling the findings to be generalisable. However, it is difficult to know whether the data that was available on breastfeeding is reliable. The quality of data may be influenced by collection methods and how often health visitors were able to visit families to ask questions about methods of feeding and how long they breastfed. Use of expressed milk and donor milk were not explored in the current study and the datasets employed in this study did not contain information regarding alternative methods. The survey will give a highly optimistic finding as the women who responded to a survey already had higher intention to breastfeed compared to the routine data population so the findings from this survey will be biased toward breastfeeding.

In conclusion, aspects of the pandemic such as working from home or time with their partner may have improved breastfeeding duration and so policies and practices that facilitate time with the family may improve duration of breastfeeding. The initiation of breastfeeding was not influenced by the pandemic so protection from infection did not appear to encourage more women to start breastfeeding. Intention to breastfeed was the

Confidential: For Review Only

strongest predictor of exclusive breastfeeding at six months and therefore intervention early in the pregnancy or before pregnancy which facilitates motivation to breastfeed may improve breastfeeding duration.

Confidential: For Review Only

Author Contributions

All authors provided substantial contributions to the piece of work. HJ, MS, MM, and AB conducting the analysis and interpretation of the data. SB conception and design of the work. HJ, MJ, and NK drafting and revising the work critically for intellectual content. All authors contributed to the final approval of the version to be published. All authors agree to be accountable for all aspects of the work.

Conflict of interest statement

There are no conflicts of interest for any of the authors.

Funding Statement

This work is funded by the National Centre for Population Health and Wellbeing Research (NCPHWR) Grant Number: AMS103836 and the National Core Studies funded by the Medical Research Council (MRC) Grant Number: MC_PC_20030.

Confidential: For Review Only

257 **References**

258 [1] Pérez-Escamilla R. Breastfeeding in the 21st century: How we can make it work.
259 *Soc. Sci. Med 2020*; 244,112331

260 [2] Feldman-Winter L. Where We Stand: Breastfeeding [Internet]. HealthyChildren.org.
261 2020. Available from: https://www.healthychildren.org/English/ages-
262 stages/baby/breastfeeding/Pages/Where-We-Stand-Breastfeeding.aspx

263 [3] Nuzzi G, Di Cicco ME, Peroni DG. Breastfeeding and Allergic Diseases: What's
264 New? Children (Basel). 2021;8(5):330. doi: 10.3390/children8050330. PMID: 33923294;
265 PMCID: PMC8145659.

266 [4] Victora CG, Bahl R, Barros AJ et al. Breastfeeding in the 21st century: epidemiology,
267 mechanisms, and lifelong effect. *Lancet* 2016;387:475–490.

268 [5] Rollins NC, Bhandari N, Hajeebhoy N et al. Why invest, and what it will take to
269 improve breastfeeding practices? *Lancet* 2016;387(10017):491-504.

270 [6] World Health Organization. Exclusive breastfeeding for six months best for babies
271 everywhere. [WHO Website]. https://www.who.int/news/item/15-01-2011-exclusive-
272 breastfeeding-for-six-months-best-for-babies-everywhere. Accessed December 13,
273 2022.

274 [7] Welsh Government. All Wales Breastfeeding Five Year Action Plan. [Welsh
275 Government Website]. https://gov.wales/sites/default/files/publications/2019-06/all-

Confidential: For Review Only

wales-breastfeeding-five-year-action-plan-july-2019_0.pdf Accessed December 13, 2022.

[8] Hunt L, Thomson G, Whittaker K, Dykes F. Adapting breastfeeding support in areas of socio-economic deprivation: a case study approach. *Int. J. Equity Health* 2021;20:83.

[9] Meedya S, Fahy K, Kable A. Factors that positively influence breastfeeding duration to 6 months: a literature review. *Women Birth* 2010;23:135–145.

[10] Vieira TO, Martins CD, Santana GS, Vieira GO, Silva LR. Intenção materna de amamentar: revisão sistemática [Maternal intention to breastfeed: a systematic review]. Cien Saude Colet. 2016;21(12):3845-3858. Portuguese. doi: 10.1590/1413-812320152112.17962015. PMID: 27925124.

[11] Linares AM, Rayens MK, Gomez ML. et al. Intention to Breastfeed as a Predictor of Initiation of Exclusive Breastfeeding in Hispanic Women. J Immigrant Minority Health. 2015;17, 1192–1198. doi.org/10.1007/s10903-014-0049-0

[12] Milani GP, Porro A, Agostoni C, Giannì ML. Breastfeeding during a Pandemic. Ann. Nutr. Metab. 2022:1-9.

[13] Vazquez-Vazquez A, Dib S, Rougeaux E, Wells JC, Fewtrell MS. The impact of the Covid-19 lockdown on the experiences and feeding practices of new mothers in the UK: Preliminary data from the COVID-19 New Mum Study. *Appetite* 2021;156,104985.

Confidential: For Review Only

294 [14] United Nations. Transforming our World: The 2030 Agenda for Sustainable
295 Development. 2015. [United Nations Website]. https://sdgs.un.org/2030agenda .
296 Accessed December 13, 2022.

297 [15] Jones KH, Ford DV, Thompson S, Lyons RA. A Profile of the SAIL Databank on the
298 UK Secure Research Platform. *IJPDS* 2019;4(2).

299 [16] Lyons RA, Ford DV, Moore L, Rodgers SE. Use of data linkage to measure the
300 population health effect of non-health-care interventions. *Lancet* 2014;383(9927):1517-
301 1519.

302 [17] Born in Wales. Expectant Parents [Born in Wales Website]. 2022.
303 https://ncphwr.org.uk/portfolio/born-in-wales-overview/ Accessed December 13, 2022.

304 [18] RStudio Team (2020). RStudio: Integrated Development for R. RStudio, PBC,
305 Boston, MA URL http://www.rstudio.com/.

306 [19] Wu Q, Tang N, Wacharasin C. Factors influencing exclusive breastfeeding for 6
307 months postpartum: A systematic review. Int. J. Nurs. Knowl. 2022;33:4:290-303.

308 [20] Rollins N, Piwoz E, Baker P, Kingston G, Mabaso KM, McCoy D. Marketing of
309 commercial milk formula: a system to capture parents, communities, science, and
310 policy. Lancet. 2023;401:10375.486-502 DOI:https://doi.org/10.1016/S0140-
311 6736(22)01931-6

312

Confidential: For Review Only

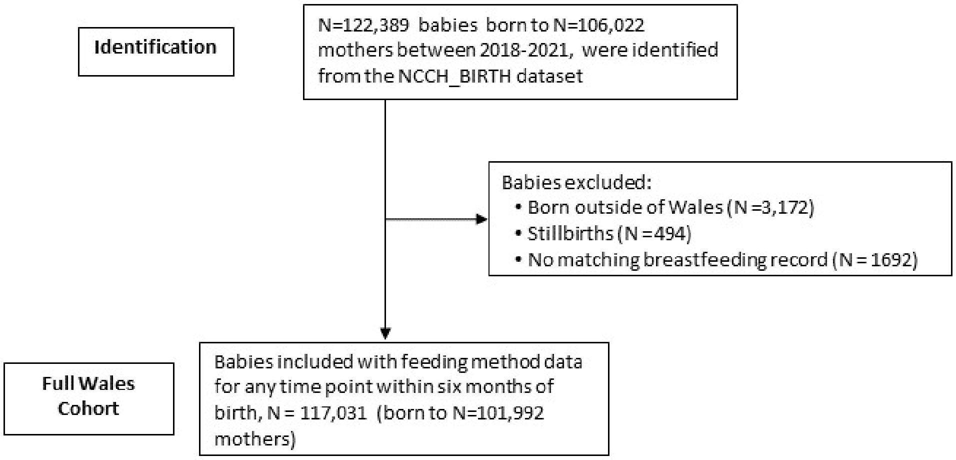

Figure 1. Study flowchart explaining how the cohort was identified

43x20mm (400 x 400 DPI)

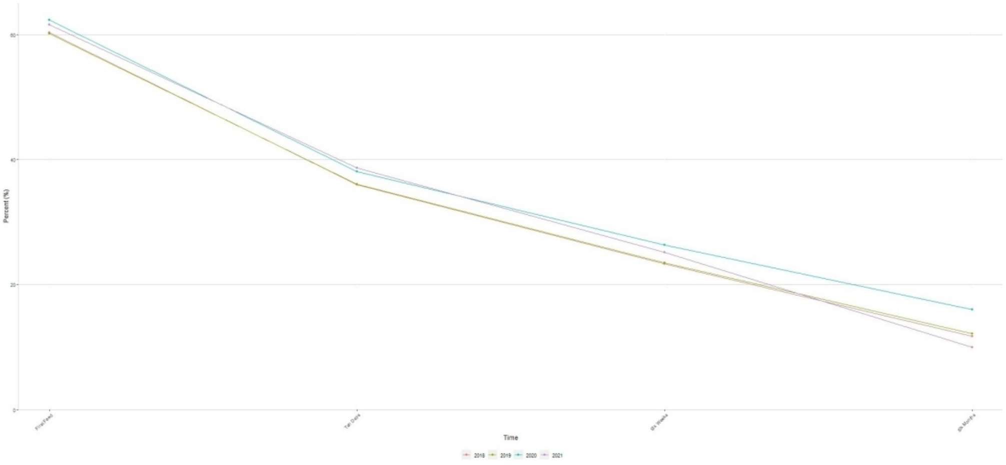

Figure 2. Exclusive breastfeeding of babies born in Wales between 2018-2021

80x37mm (600 x 600 DPI)

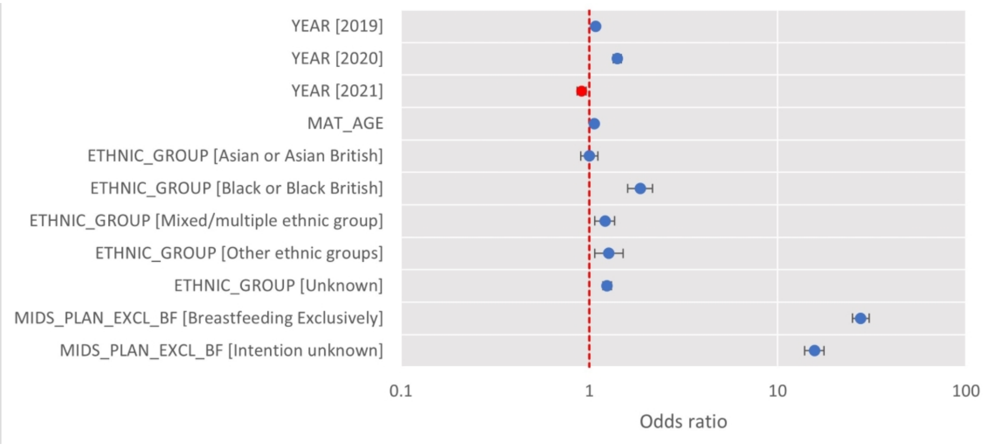

Figure 3. Multinomial regression for exclusive breastfeeding for six months

140x61mm (400 x 400 DPI)
